# Force-Induced Autophagy in Periodontal Ligament Stem Cells Modulates M1 Macrophage Polarization via AKT Signaling

**DOI:** 10.3389/fcell.2021.666631

**Published:** 2021-05-26

**Authors:** Nan Jiang, Danqing He, Yushi Ma, Junxiang Su, Xiaowen Wu, Shengjie Cui, Zixin Li, Yanheng Zhou, Huajie Yu, Yan Liu

**Affiliations:** ^1^Central Laboratory, Peking University School and Hospital of Stomatology & National Engineering Laboratory for Digital and Material Technology of Stomatology & Beijing Key Laboratory of Digital Stomatology, Beijing, China; ^2^Laboratory of Biomimetic Nanomaterials, Department of Orthodontics, Peking University School and Hospital of Stomatology & National Engineering Laboratory for Digital and Material Technology of Stomatology & Beijing Key Laboratory of Digital Stomatology, Beijing, China; ^3^Department of Endodontics, Shanxi Medical University School and Hospital of Stomatology, Shanxi, China; ^4^The Fourth Division, Peking University School and Hospital of Stomatology, Beijing, China

**Keywords:** autophagy, macrophage polarization, bone remodeling, mechanical force, periodontal ligament stem cells, inflammation, tooth movement, AKT signaling

## Abstract

Autophagy, a lysosomal degradation pathway, serves as a protective cellular mechanism in maintaining cell and tissue homeostasis under mechanical stimulation. As the mechanosensitive cells, periodontal ligament stem cells (PDLSCs) play an important role in the force-induced inflammatory bone remodeling and tooth movement process. However, whether and how autophagy in PDLSCs influences the inflammatory bone remodeling process under mechanical force stimuli is still unknown. In this study, we found that mechanical force stimuli increased the expression of the autophagy protein LC3, the number of M1 macrophages and osteoclasts, as well as the ratio of M1/M2 macrophages in the compression side of the periodontal ligament *in vivo*. These biological changes induced by mechanical force were repressed by the application of an autophagy inhibitor 3-methyladenine. Moreover, autophagy was activated in the force-loaded PDLSCs, and force-stimulated PDLSC autophagy further induced M1 macrophage polarization *in vitro.* The macrophage polarization could be partially blocked by the administration of autophagy inhibitor 3-methyladenine or enhanced by the administration of autophagy activator rapamycin in PDLSCs. Mechanistically, force-induced PDLSC autophagy promoted M1 macrophage polarization via the inhibition of the AKT signaling pathway. These data suggest a novel mechanism that force-stimulated PDLSC autophagy steers macrophages into the M1 phenotype via the AKT signaling pathway, which contributes to the inflammatory bone remodeling and tooth movement process.

## Introduction

Mechanical force plays a vital role in maintaining tissue homeostasis and mediating pathological process under physiological or pathological conditions (Smutny et al., 2017; Miroshnikova et al., 2018). In the alveolar bone environment, mechanical force supports the homeostasis during masticatory movements (Thompson et al., 2012), and also mediates the bone remodeling process during tooth movement (Meikle, 2006). During tooth movement, aseptic inflammatory microenvironment is developed in the periodontal tissues, which is characterized by elevated expressions of inflammatory cytokines, chemokines, and increased activations of inflammatory immune cells (Garlet et al., 2007; He et al., 2015b; Yan et al., 2015). As the main mesenchymal stem cells (MSCs) in the periodontal tissues, periodontal ligament stem cells (PDLSCs) constantly receive mechanical force stimuli and contribute to the inflammatory responses and bone remodeling process during tooth movement (Zhang et al., 2016; Huang et al., 2018). Increased expressions of inflammatory cytokines, chemokines, and gas molecules such as hydrogen sulfide have been found in the force-stimulated PDLSCs (Lee et al., 2012; Liu et al., 2017; He et al., 2020). However, how mechanical force modulates PDLSC behaviors and therefore influences the inflammatory responses and the bone remodeling process under force stimuli is still obscure.

Autophagy has been gradually acknowledged as an important protective cellular process to maintain cell and tissue homeostasis under the external stimuli, such as stress, inflammation, hypoxia, and mechanical load (Hara et al., 2006; Jiang et al., 2010; Kroemer et al., 2010). Cells could degrade damaged organelles and misfolded proteins, and therefore maintain themselves survival. The degraded components are encapsulated in autophagic vacuoles or autophagosomes, which fuse with lysosomes to form autophagolysosomes (Mizushima et al., 2008; Klionsky et al., 2012). Autophagy has also been shown to participate in the pathological process of inflammatory diseases, such as chronic intestinal inflammation, inflammatory bowel diseases and inflammatory periodontitis (Levine et al., 2011; An et al., 2016; Wang et al., 2019). In the treatment of autoimmune encephalomyelitis, autophagy has been discovered to modulate the immunoregulatory properties of MSCs (Dang et al., 2014). In addition, autophagy might also be adapted to mechanical load, which could be activated in mechanosensitive cells such as osteoblasts, endothelial cells, and PDLSCs (King et al., 2011; Ma et al., 2013; Memmert et al., 2019). However, whether autophagy in PDLSCs influenced inflammatory microenvironment of the periodontal tissues under mechanical force stimuli needs further exploration.

During the force-induced inflammatory bone remodeling process, macrophages are regarded as one of the vital immune cells (Feng and Teitelbaum, 2013; Horwood, 2016). Depending on the different environmental signals, macrophages show a broad spectrum of activation phenotypes, described by M1 or M2 polarization. The M1 phenotype mainly mediates the inflammation process. It could be activated by interferon (IFN)-γ or lipopolysaccharides, and expressed inflammatory elements such as tumor necrosis factor (TNF)-α and nitric oxide (NO); the M2 phenotype mainly participates in tissue remodeling, which could be activated by interleukin (IL)-4 or IL-13 and could produce IL-10, arginase-1 (Arg-1) and DECTIN-1 (Murray, 2017; Shapouri-Moghaddam et al., 2018). Previously, we have confirmed that M1 macrophage polarization is critical in the bone remodeling and root resorption process during tooth movement (He et al., 2015a,b). Given the importance of M1 macrophage polarization during tooth movement, we hypothesized that autophagy in PDLSCs influences macrophage polarization under mechanical force stimuli and thus influences alveolar bone remodeling. This study aims to verify whether and how force-induced autophagy in PDLSCs modulates macrophage polarization and contributes to periodontal inflammatory microenvironment and bone remodeling during tooth movement.

## Materials and Methods

### Animals and Treatments

Adult Sprague-Dawley rats (180–200 g, 6–8 weeks, male) were divided into three groups, including the group with force loading (Force, *n* = 10), the group with force loading and 3-methyladenine application (Force + 3-MA, *n* = 10) and the control group without force loading (Con, *n* = 10). Orthodontic force was applied for 7 days as previously described (He et al., 2015b). Briefly, a nickel–titanium coil spring (wire size 0.2 mm, 1 mm in diameter, and 1 mm in length, Smart Technology, China) was bonded between the upper first molar and incisors of rats to provide approximately 50–60 g force (Taddei et al., 2012). Volumetrically equivalent saline or 3-MA (30 mg/kg, Sigma, St. Louis, MO, United States) was injected intraperitoneally every day during tooth movement (Jiang et al., 2010) in the Force or Force + 3-MA group, respectively.

The rats were sacrificed after 7 days, and the maxillae were harvested for further studies. The tooth movement distance was measured between the midpoint of the distal-marginal ridge of the maxillary first molar and the midpoint of the mesial-marginal ridge of the maxillary second molar (He et al., 2015b). The measurement was repeated for three times and the average distance was calculated. All experimental protocols were approved by the Institutional Animal Use and Care Committee of Peking University (LA2013-92).

### Human PDLSCs Culture and Mechanical Force Loading

Human PDLSCs were isolated from the extracted healthy premolars of volunteers receiving orthodontic treatment with informed consent as previously described (Seo et al., 2004). The ethic protocols were approved by the Peking University Ethical Committee (PKUSSIRB-201311103). Briefly, the periodontal ligament was scraped from the root surface and minced into small pieces. Periodontal tissue explants were cultured in cell culture flask upside down with DMEM medium (Invitrogen, Carlsbad, CA, United States) with 20% fetal bovine serum (Gibco, MA, United States) and 1% Penicillin/Streptomycin. Cells were grown in a CO_2_ incubator at 37°C. The PDLSCs were identified following our previous protocols (Fu et al., 2016) and used at passage four for further analysis.

To apply compressive force on PDLSCs, glass layers and 15 ml plastic tube caps containing weighed metal balls were used to apply compressive force (0.5–2.5 g/cm^2^) lasting for different time points, which was modified from a previously described method (Kanzaki et al., 2002). After subjected to compressive force for 12 h, PDLSCs were collected for further experiments of transmission electronic microscopy (TEM), autophagy flux assay and immunofluorescence staining. Next, the culture medium from force-loaded PDLSCs (1.5 g/cm^2^, 12 h) and force-loaded PDLSCs with additional treatment of 3-MA (2.5 mM, 12 h) or rapamycin (0.25 μg/ml, 12 h, Sigma, St. Louis, MO, United States) (Ma et al., 2018) were collected to treat THP-1-derived macrophages.

### Transmission Electronic Microscopy

Periodontal ligament stem cells with or without force loading were fixed in 2.5% (v/v) glutaraldehyde, and treated with 3% potassium ferrocyanide and 1% (w/v) osmium tetroxide for 1 h at 4°C. Cells were then treated with 0.3% thiocarbohydrazide for 5 min at room temperature, and then incubated in 1% osmium tetroxide for 20 min at 4°C. After dehydrated in the series of ethanol, cells were embedded and the ultra-thin sections were harvested and stained with solution containing 1% toluidine blue and 2% borate. Images were observed with a JEM-1400-Plus transmission electron microscope (JEOL, Japan).

### Autophagy Flux Assays

Periodontal ligament stem cells were transfected with the mRFP-GFP-LC3 adenoviral (HanBio, Shanghai, China) according to the manufacturer’s instructions. After infection, cells were cultured for another 24 h and then subjected to compressive force (1.5 g/cm^2^) for 12 h. After force loading, autophagosomes and autolysosomes were observed and captured with a laser scanning microscope (LSM 510, Zeiss, Jena, Germany) and the images were processed using LSM 5 Release 4.2 software. The intensity of autophagy flux was determined by assessing the numbers of RFP/GFP-expressing cells.

### Treatment of THP-1-Derived Macrophages

THP-1 human monocytic cells (ATCCTIB-202) were differentiated into macrophages with the treatment of 50 ng/ml phorbol 12-myristate 13-acetate (PMA, Sigma) for 24 h. To determine whether the autophagy of force-stimulated PDLSCs could influence macrophage polarization, THP-1-derived macrophages were incubated with supernatant from the force-loaded PDLSCs (FS) and force-loaded PDLSCs with additional treatment of 3-MA (FS + 3-MA) or rapamycin (FS + Rapa). THP-1-derived macrophages incubated with supernatant from the PDLSCs without force-loaded served as control (CS). After 24 h, total RNA and total protein were harvested from THP-1-derived macrophages for further detection of macrophage-related markers.

To further explore the underlying mechanism, total protein was harvested from THP-1-derived macrophages after incubation in the conditioned medium for 2-4 h to detect the changes of signaling pathway. Next, the activator of AKT signaling pathway IGF1 (100 ng/ml, Abcam, United States) was applied to the macrophages of FS + Rapa group, and the inhibitor of AKT singnalling pathway GSK690693 (10 μM, Beyotime Biotechnology, Jiangsu, China) was applied to the macrophages of FS + 3-MA group. Total protein was extracted at 30 min to test the chemical drug efficiency. In addition, total RNA and protein were harvested from THP-1-derived macrophages after 24 h treatment for further detection of macrophage-related markers.

### Immunohistochemical Staining

The trimmed maxillae were fixed in 10% neutral buffered formalin for 24 h, decalcified in 15% ethylenediaminetetraacetic acid and embedded in paraffin. Consecutive 4-μm sagittal sections of the maxillary first molar were obtained and the middle to apical third of the periodontal tissues was observed. A two-step detection kit (Zhongshan Golden Bridge Biotechnology, Beijing, China) was used following previous protocols (He et al., 2015b). Primary antibodies including anti-microtubule associated protein 1 light chain 3 β (LC3B) (1:100, CST3868S, CST), anti-TNF-α (1:100, ab1793, Abcam), and anti-CD146 (1:200, ab-75769, Abcam) were used. Five different slides from each sample (*n* = 10) were used for cell counting. Each slide was measured for three times and the average positive cell numbers were calculated.

### Tartrate-Resistant Acid Phosphatase Staining

Histology sections were stained with an acid phosphatase kit (387A-1KT; Sigma) for tartrate-resistant acid phosphatase (TRAP) staining. TRAP-positive multinucleated cells (≥3 nuclei) attached to the surface of the adjacent alveolar bone were counted as osteoclasts.

### Immunofluorescence Staining

For immunofluorescence staining, tissue sections were double-stained with anti-CD68 (1:500; MCA341GA, Serotec, United Kingdom) and anti-inducible nitric oxide synthase (iNOS) (1:100; ab-15323, Abcam) for M1 macrophages, or anti-CD68 and anti-CD163 (1:100; sc-33560, Santa Cruz) for M2 macrophages. PDLSCs were stained with anti-LC3B (1:100, CST3868S, CST). Samples were incubated with primary antibodies at 4°C overnight. On the following day, the samples were incubated with fluorescein secondary antibody (1:200, Jackson Immuno Research Laboratories, West Grove, PA, United States). Nuclei were counterstained with 4’,6-diamidino-2-phenylindole (DAPI). Samples were observed with a laser scanning microscope (LSM 510, Zeiss), and the images were processed using LSM 5 Release 4.2 software. Three different slides from each sample (*n* = 6) were used for cell counting. Each slide or cell sample was measured for three times and the average positive cell numbers were calculated.

### Quantitative Real-Time Polymerase Chain Reaction

Total RNA was extracted using TRizol reagent (Invitrogen, Carlsbad, CA, United States) in accordance with the manufacturer’s instructions. RNA samples (2000 ng) were reverse transcribed into cDNA using RevertAid First Strand cDNA Synthesis kit (Thermo Fisher Scientific). Then real-time Polymerase Chain Reaction (PCR) was performed using the FastStart Universal SYBR Green master kit (Roche) on an Applied Biosystems 7500 Real-Time PCR System (Life Technologies Corporation, United States) to determine the relative mRNA expression level of autophagy marker Beclin-1. M1 macrophage markers including TNF-α, iNOS, and M2 macrophage markers including arginase-1 (Arg-1) and DECTIN-1 were also detected. Fold changes of target genes were calculated with △△CT method using GAPDH as reference control. The sequences of human primers (designed by Primer Premier 5.0 software) were listed as follows:

GAPDH sence/antisence: 5′-ATGGGGAAGGTGAAGGT CG-3′/5′-GGGGTCATTGATGGCAACAATA-3′.
TNF-α sence/antisence: 5′-GAGGCCAAGCCCTGGTA TG-3′/5′-CGGGCCGATTGATCTCAGC-3′.
iNOS sence/antisence: 5′-TTCAGTATCACAACCTCAGC AAG-3′/5′-TGGACCTGCAAGTTAAAATCCC-3′.
DECTIN-1 sence/antisence: 5′-GGAAGCAACACATTGG AGAATGG-3′/5′-CTTTGGTAGGAGTCACACTGTC-3′.
arginase-1 sence/antisence: 5′-TGGACAGACTAGGAATT GGCA-3′/5′-CCAGTCCGTCAACATCAAAACT-3′.
Beclin-1 sence/antisence: 5′-ATTCGAGAGCAGCATCC AAC-3′/5′-AACAGGAAGCTGCTTCTCAC-3′.

### Western Blot Analysis

Western blot analyses were performed following previous protocols (He et al., 2015b). Total protein was extracted from cultured cells using RIPA lysis buffer (Sigma) with protein inhibitor cocktail (Roche) and PhosSTOP Phosphatase inhibitor cocktail (Roche, United States). Lysates were centrifuged at 12,000 × *g* for 15 min. Supernatants were collected for further use. Proteins were separated by 10% SDS-PAGE, transferred onto PVDF membrane (Bio-Rad), blocked in 5% BSA and then probed overnight with primary antibodies included anti-β-actin (1:10,000, a-5441, Sigma), anti-GAPDH (1:10,000, 60004, Proteintech), anti-LC3(1:500, CST3868S, CST), anti-P62 (1:1,000, ab91526, Abcam), anti-iNOS (1:500, abs130136, Absin), anti-Arg-1 (1:5,000, sc-21050, Santa Cruz), anti-TNF-α (1:200, sc-52746, Santa Cruz), anti-phospho-AKT1 (Ser473) (1:500, 9018S, CST), anti-AKT (1:500, 9272, CST), and anti-NF-κB/P65 (1:1,000, 8242S, CST). The blots were then incubated with a horseradish peroxidase-conjugated secondary antibodies (1:5,000; Zhongshan Golden Bridge Biotechnology, Beijing, China). Finally, the protein bands were enhanced by chemiluminescence detection before photography. The relative density of at least three comparable results was measured by Image J software.

### Statistical Analysis

Data were presented as mean ± SD. Student’s *t*-tests or one-way analysis of variance (ANOVA) with *post hoc* tests were used to compare the differences between groups using SPSS20.0. *P* < 0.05 was considered statistically significant.

## Results

### Force-Induced Autophagy in PDLSCs Contributes to M1 Macrophage Polarization *in vivo*

**FIGURE 1:**
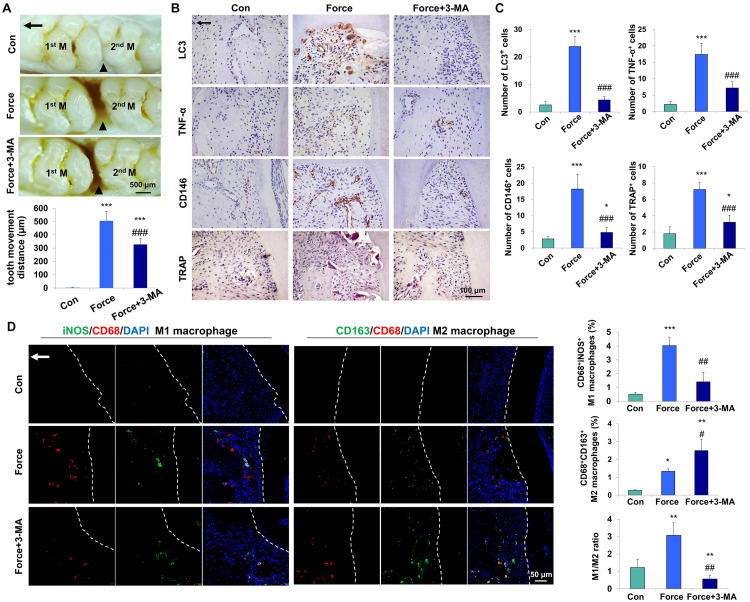
Mechanical force-induced autophagy contributes to M1 macrophage polarization and bone remodeling during tooth movement *in vivo*. **(A)** Representative images and semiquantification analysis of the tooth movement distance in rats. Force loading increased the tooth movement distance, which was partially reversed by 3-MA application. 1st M, the first molar; 2nd M, the second molar. Black triangles represent the tooth movement distance. **(B)** Representative immunohistochemical images of LC3, TNF-α and CD146 and TRAP staining. **(C)** Semiquantification analysis of the number of LC3^+^, TNF-α^+^ and CD146^+^ and TRAP^+^ cells in panel **(B)**. **(D)** Representative immunofluorescence images on the compression side of distobuccal roots and semiquantification analysis of double-labeled cells. M1 macrophage polarization was identified by CD68^+^ (red) and inducible nitric oxide synthase (iNOS)^+^ (green), whereas M2 macrophage polarization was identified as CD68^+^ (red) and CD163^+^ (green). Dashed lines mark the outline of distobuccal roots. Arrow represents the direction of force. Results are presented as mean ± SD. *n* = 10. *P < 0.05, **P < 0.01, ***P < 0.001 versus Con; ^#^*P* < 0.05, ^##^*P* < 0.01, ^###^*P* < 0.001 versus Force.

To investigate whether mechanical force could activate autophagy, an experimental animal model of tooth movement was set up and autophagy was systemically blocked with the inhibitor 3-MA. After force loading for 7 days, the tooth movement distance increased to 505.4 ± 72.4 μm, and was partially reversed to 327.8 ± 47.27 μm after 3-MA injection (*P* < 0.001, Figure 1A). The expressions of the autophagy protein LC3, the pro-inflammatory cytokine TNF-α and the MSC surface marker CD146 increased in the compression side of the periodontal tissues after force loading (*P* < 0.001). Concomitantly, the number of TRAP^+^ osteoclasts also increased after force loading (*P* < 0.001, Figures 1B,C). 3-MA injection significantly decreased the expressions of LC3, TNF-α and CD146, as well as the number of TRAP^+^ osteoclasts (*P* < 0.001). Nevertheless, the number of CD146^+^ MSCs and TRAP^+^ osteoclasts was still upregulated in the Force + 3-MA group, compared with the control group (*P* < 0.05, Figures 1B,C).

Mechanical force modulates M1 macrophage polarization, which contributes to bone remodeling and root resorption during tooth movement (He et al., 2015b). To assess the influence of force-induced autophagy on macrophage polarization *in vivo*, immunofluorescence staining was performed to identify the changes of macrophage markers after force loading. The number of CD68^+^iNOS^+^ M1 macrophages increased on the compression side of the periodontal ligament after force loading (*P* < 0.001 versus Con) and decreased significantly after 3-MA injection (*P* < 0.01 versus Force). Although the number of CD68^+^CD163^+^ M2 macrophages increased after force loading and 3-MA injection (*P* < 0.05 and 0.01, respectively), the ratio of M1/M2 macrophage polarization increased significantly after force loading (*P* < 0.01 versus Con) and decreased significantly after 3-MA injection (*P* < 0.01 versus Con and Force, Figure 1D). These data suggest that autophagy modulates macrophage polarization after force loading and influences the alveolar bone remodeling and tooth movement process.

### Mechanical Force Activates Autophagy in PDLSCs

**FIGURE 2:**
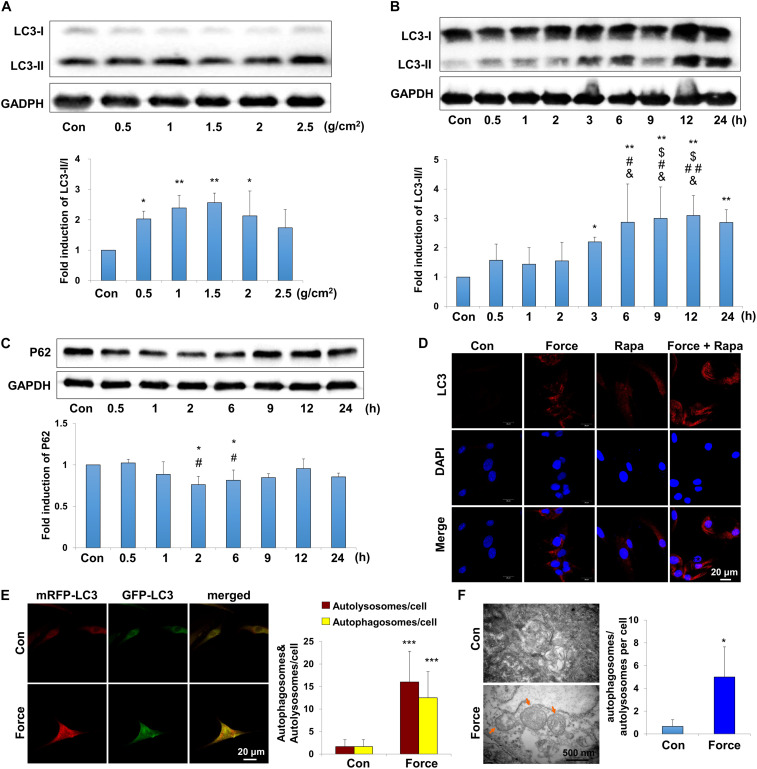
Autophagy is induced with the application of mechanical stimuli in PDLSCs. **(A)** Western blot of the expressions of LC3 in PDLSCs under different force values at protein level. The relative fold change of LC3-II/I was quantified. GAPDH served as an internal control for equal loading. Results are presented as mean ± SD. *n* = 3–4. ^∗^*P* < 0.05, ^∗∗^*P* < 0.01 versus Con. **(B)** Western blot of the expressions of LC3 in PDLSCs under force stimuli (1.5 g/cm^2^) at different time points. The relative fold change of LC3-II/I was quantified. GAPDH served as an internal control for equal loading. Results are presented as mean ± SD. *n* = 3–4. ^∗^*P* < 0.05, ^∗∗^*P* < 0.01 versus Con;^$*I*^*P* < 0.05 versus 0.5 h; ^#^*P* < 0.05,^##^*P* < 0.01 versus 1 h; ^&^*P* < 0.05, ^&&^*P* < 0.01 versus 2 h. **(C)** Western blot and quantification of P62 expression in PDLSCs under force stimuli (1.5 g/cm^2^) at different time points. Results are presented as mean ± SD. *n* = 3. ^∗^*P* < 0.05 versus Con; ^#^*P* < 0.05 versus 0.5 h. **(D)** Immunofluorescence staining of LC3 (red) in PDLSCs with the application of force stimuli (1.5 g/cm^2^) or rapamycin (Rapa). **(E)** PDLSCs were infected with adenovivus with mRFP-GFP-LC3. Cells were applied with static force for 6 h, and the autophagosomes and autolysosomes were detected in PDLSCs. Yellow-colored autophagosomes and red-colored autolysosomes were calculated. Results are presented as mean ± SD. *n* = 4. ^∗∗∗^*P* < 0.001 versus Con. **(F)** Ultrastructural features in PDLSC assessed by TEM without or with force. Arrows indicated the autophagosomes or autolysosomes in the cytoplasm. Numbers of autophagosomes/autolysosomes were quantified. Results are presented as mean ± SD. *n* = 3. ^∗^*P* < 0.05 versus Con.

Periodontal ligament stem cells, as the main MSCs in the periodontal ligament microenvironment, respond to mechanical force and participate in the alveolar bone remodeling and tooth movement. To explore the autophagy activity under mechanical force, we performed western blot, immunofluorescence staining autophagy flux assay and TEM on PDLSCs *in vitro*. LC3 is one of the most essentially monitored autophagy-related proteins. Increased expression of LC3II/I was detected under compressive force loading from 0.5 to 2.5 g/cm^2^ for 12 h. Nevertheless, 1.0 and 1.5 g/cm^2^ compressive force triggered the strongest expressions of LC3II/I (Figure 2A), and 1.5 g/cm^2^ compressive force was utilized in the following experiments. In addition, the expressions of LC3II/I started to increase increased after force stimulation for 3 h and persisted to 24 h (Figure 2B). Moreover, the expression of another autophagy marker P62/SQSTM (P62) decreased after compressive force loading for 2 and 6 h (*P* < 0.05, Figure 2C); whereas the expression of Beclin1 increased after force stimuli for 3 h (*P* < 0.05, Supplementary Figure 1A). Accordingly, real-time PCR showed that the mRNA expression of Beclin1 was significantly upregulated compared with the control group (*P* < 0.01, Supplementary Figure 1B). Immunofluorescence staining showed that positive expression of LC3 aggregated in PDLSCs after compressive force stimulation for 12 h, which was further enhanced after rapamycin application (Figure 2D). Autophagosomes, also known as initial autophagic vacuoles, are spherical structures with bilayers membranes containing cytoplasmic components and/or organelles (Kliosnky et al., 2016). mRFP-GFP-LC3 adenovirus were transfected into PDLSCs to detect autophagic flux. Cells were applied with compressive force for 12 h, and the flux rate of autophagy was detected with microscopy. The numbers of autolysosomes (mRFP-positive, red dots) and autophagosomes (GFP/RFP double-positive, yellow dots) were significantly higher in the force group (*P* < 0.001, Figure 2E). In addition, autophagosomes could be obviously identified in the PDLSCs by TEM after compressive force stimulation (Figure 2F). Taken together, these data reveal that under compressive force stimulation, autophagy can be activated in a force-dependent and time-dependent tendency in PDLSCs.

### Force-Stimulated PDLSC Autophagy Induces M1 Macrophage Polarization *in vitro*

**FIGURE 3:**
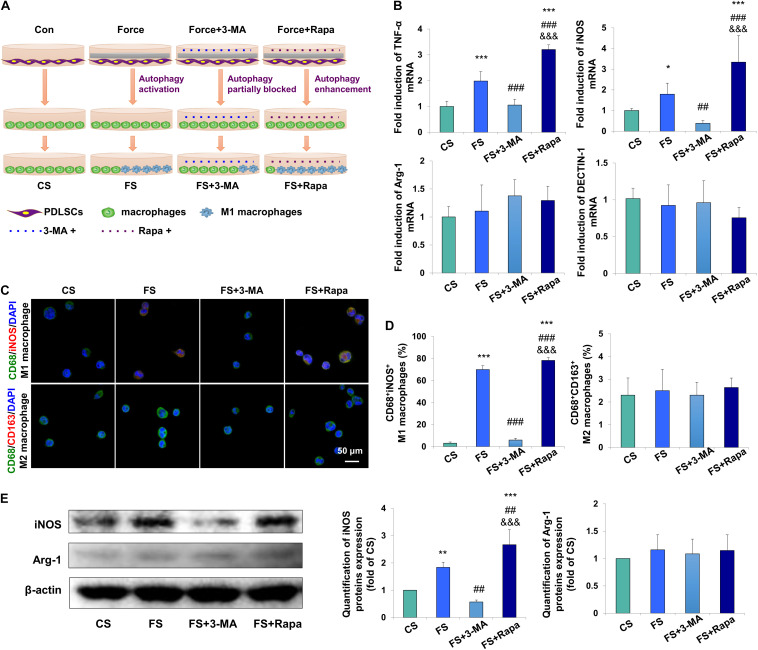
Force-stimulated PDLSC autophagy induces M1 macrophage polarization *in vitro*. **(A)** Schematic illustration. **(B)** Relative mRNA expression of M1/M2-related genes of THP-1-derived macrophages with treatment of different conditioned medium. CS: control supernatant; FS: force-loaded supernatant. FS + 3-MA: force-loaded supernatant with 3-MA; FS + Rapa: force-loaded supernatant with rapamycin. Results are presented as mean ± SD. *n* = 6.^*^P < 0.05, ^∗∗^*P* < 0.01, ^∗∗∗^*P* < 0.001 versus CS; ^##^*P* < 0.01, ^###^*P* < 0.001 versus FS; ^&&&^*P* < 0.001 versus FS + 3-MA. **(C)** Representative immunocytochemical images of THP-1-derived macrophages. M1 macrophage polarization [CD68^+^ (green) and iNOS^+^ (red)] increased in the FS group, which decreased significantly after 3-MA application or enhanced after rapamycin application. No change was observed in M2 macrophage polarization [CD68^+^ (green) and CD163^+^ (red)]. **(D)** Quantification of CD68^+^/iNOS^+^ double positive cells and CD68^+^/CD163^+^ double positive cells in panel **(C)**. Results are presented as mean ± SD. *n* = 3–4. ^∗∗∗^*P* < 0.001 versus CS; ^###^*P* < 0.001 versus FS; ^&&&^*P* < 0.001 versus FS + 3-MA. **(E)** Western blot of the expressions of iNOS and arginase-1 in THP-1-induced macrophages. Beta-actin served as an internal control for equal loading. Protein expression level was quantified and presented as mean ± SD. *n* = 3. ^∗∗^*P* < 0.01, ^∗∗∗^*P* < 0.001 versus CS; ^##^*P* < 0.01 versus FS; ^&&&^*P* < 0.001 versus FS + 3-MA.

To further detect whether autophagy in force-stimulated PDLSCs could affect macrophage polarization, the supernatant from the force-loaded PDLSCs (FS), the force-loaded PDLSCs with additional treatment of an autophagy inhibitor 3-MA (FS + 3-MA) or an autophagy activator rapamycin (FS + Rapa) was utilized to treat THP-1-derived macrophages. THP-1-derived macrophages incubated with supernatant from PDLSCs without force loading served as the control (CS) (Figure 3A).

The mRNA expression levels of M1 macrophage associated markers TNF-α and iNOS in THP-1-derived macrophages were upregulated in the FS group (*P* < 0.001 and *P* < 0.05 versus CS, respectively), which were downregulated in the FS + 3-MA group (*P* < 0.001 and *P* < 0.01 versus FS, respectively) and further enhanced in the FS + Rapa group (*P* < 0.001 versus CS, FS and FS + 3-MA). However, no changes were observed of the mRNA expressions of M2 macrophage associated markers arginase-1 and DECTIN-1 (Figure 3B).

Immunocytochemical analyses showed that the proportion of CD68^+^iNOS^+^ M1 macrophages increased significantly in the FS group (*P* < 0.001), which was partially blocked in the FS + 3-MA (*P* < 0.001) group and enhanced in the FS + Rapa group (*P* < 0.001). However, the proportion of CD68^+^CD163^+^ M2 macrophages did not changed [Figures 3C,D and Supplementary Figure 2 (Split images)]. Correspondingly, western blot analysis showed that the expression of M1 macrophage associated marker iNOS significantly increased in the FS group (*P* < 0.01 versus CS), which was downregulated in the FS + 3-MA group (*P* < 0.01 versus FS) and upregulated in the FS + Rapa group (*P* < 0.001 versus CS, *P* < 0.01 versus FS and *P* < 0.001 versus FS + 3-MA, Figure 3E). The same changes of TNF-α expression were detected (Supplementary Figure 3). However, the expression of M2 macrophage associated marker arginase-1 did not changed (Figure 3E). The application of 3-MA or rapamycin exclusively did not change the expressions of TNF-α or arginase-1 in THP-1 macrophages (Supplementary Figure 4). Taken together, these data suggest that autophagy in force-loaded PDLSCs could steer macrophage polarization toward the M1 phenotype *in vitro*.

### Periodontal Ligament Stem Cell Autophagy Modulates M1 Macrophage Polarization Through the Inhibition of the AKT Signaling Pathway

**FIGURE 4:**
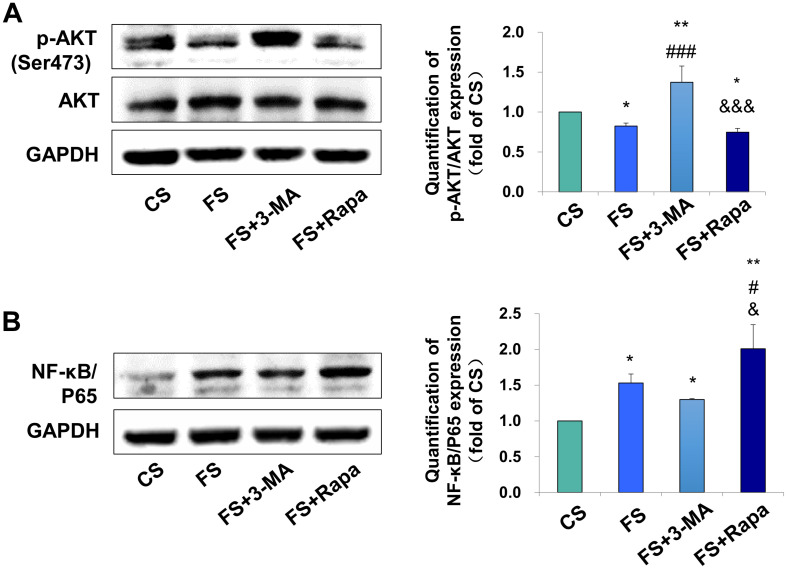
Autophagy in PDLSCs under force stimuli suppressed AKT signaling in THP-1-derived macrophages. **(A)** Western blot of the expressions of phosphor-AKT (Ser473) and AKT in THP-1-derived macrophages. GAPDH served as an internal control. The relative fold change of p-AKT/AKT was quantified. Activation of autophagy could reduce active AKT activity in FS and FS + Rapa groups, while 3-MA (FS + 3-MA) reversed these effects. **(B)** Western blot of the expressions of NF-κB/P65 in THP-1-derived macrophages. GAPDH served as an internal control. The expression of NF-κB/P65 increased after force loading, which further enhanced after rapamycin application. The relative fold change was quantified. Results are presented as mean ± SD. *n* = 3. *P < 0.05, **P < 0.01 versus CS; ^#^*P* < 0.05, ^###^*P* < 0.001 versus FS; ^&^*P* < 0.05, ^&&&^*P* < 0.001 versus FS + 3-MA.

After verifying the relationship between PDLSC autophagy and macrophage polarization, we next explored the possible underlying mechanism. AKT signaling has been acknowledged as a crucial mediator of the macrophage survival and polarization (Vergadi et al., 2017) and NF-κB is one of the key downstream signal of the AKT signaling. As a critical transcriptional factor in macrophages, increased NF-κB activity steers macrophage polarization toward the M1 phenotype (Kono et al., 2014; Hoover et al., 2020). We found the expression of phospho-AKT in macrophages was significantly inhibited in the FS groups (*P* < 0.05 versus CS) when applying the conditioned medium into the THP-1-derived macrophages, whereas blocking autophagy by 3-MA (FS + 3-MA) increased the expression of phospho-AKT (*P* < 0.01 versus CS, *P* < 0.001 versus FS) and enhancing autophagy by rapamycin reversed the above-mentioned increase level of phospho-AKT (*P* < 0.05 versus CS, *P* < 0.001 versus FS + 3-MA, Figure 4A). In contrast, the expression of NF-κB/P65 was upregulated after incubation in the supernatant from the force-loaded PDLSCs (*P* < 0.05 versus CS), which was further enhanced by the application of autophagy activator rapamycin (*P* < 0.01 versus CS, *P* < 0.05 versus FS and FS + 3-MA, Figure 4B).

**FIGURE 5:**
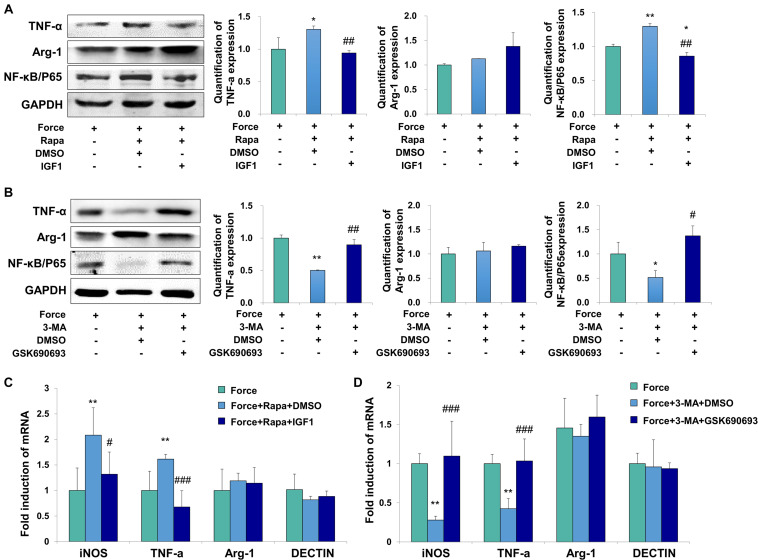
Force induced autophagy in PDLSCs modulates M1 macrophage polarization through AKT signaling. **(A)** Western blot of the expressions of TNF-α, Arg-1 and NF-κB/P65 by the application of force-loaded supernatant (FS) with rapamycin. AKT signaling activator IGF1 or equal volume of DMSO was added into supernatant. The protein levels were quantified. Results are presented as mean ± SD. *n* = 3. *P < 0.05, **P < 0.01 versus the left column. ^##^*P* < 0.01 versus middle column. **(B)** Western blot of the expressions of TNF-α, Arg-1 and NF-κB/P65 by the application of force-loaded supernatant (FS) with 3-MA. AKT kinase inhibitor GSK690693 or equal volume of DMSO was added into conditioned medium. Results are presented as mean ± SD. *n* = 3. *P < 0.05, **P < 0.01 versus the left column. ^#^*P* < 0.05, ^##^*P* < 0.01 versus middle column. **(C)** Relative mRNA expression of M1/M2-related genes of THP-1-derived macrophages cultured with different conditions, including FS, FS + Rapa + DMSO and FS + Rapa + IGF1. **(D)** Relative mRNA expression of M1/M2-related genes of THP-1-derived macrophage cultured with different conditions, including FS, FS + 3 − MA + DMSO and FS + 3 − MA + GSK690693 groups. Results are presented as mean ± SD. *n* = 3–4. **P < 0.01 versus left column; ^#^*P* < 0.05, ^###^*P* < 0.001 versus middle column.

To further confirm the regulatory mechanism, the AKT signaling activator IGF1 and inhibitor GSK690693 were applied to the THP-1-derived macrophages. The activator efficiency of IGF1 was confirmed by the increased expression of phospho-AKT/AKT (*P* < 0.01, Supplementary Figure 5A), whereas the inhibitor efficiency of GSK690693 was confirmed by the decreased expression of phospho-AKT/AKT (*P* < 0.05, Supplementary Figure 5B). The expressions of TNF-α and NF-κB/P65 increased in FS + Rapa + DMSO group (*P* < 0.05 and *P* < 0.01 versus FS), which was decreased after AKT activator IGF1 application (*P* < 0.01 versus FS + Rapa + DMSO, Figure 5A). No changes were observed of the expression the M2 marker arginase-1. Consistently, the mRNA expression levels of M1 macrophage-related markers iNOS and TNF-α increased in FS + Rapa + DMSO group (*P* < 0.01 versus FS) and decreased after IGF1 application (*P* < 0.05 and 0.001 versus FS + Rapa + DMSO), whereas no changes were observed in the expressions of M2 macrophage-related markers arginase-1 and DECTIN-1 (Figure 5C).

Furthermore, the expressions of TNF-α and NF-κB/P65 decreased in FS + 3-MA + DMSO group (*P* < 0.01 and 0.05 versus FS). After the application of AKT kinase inhibitor GSK690693, the expressions of TNF-α and NF-κB/P65 increased significantly at protein level (*P* < 0.01 and 0.05 versus FS + 3-MA + DMSO, Figure 5B). Consistently, the mRNA expression levels of iNOS and TNF-α decreased in FS + 3-MA + DMSO group (*P* < 0.01 versus FS) and increased after GSK690693 application (*P* < 0.001 versus FS + 3-MA + DMSO), whereas no changes were observed in the expressions of M2 macrophage-related markers arginase-1 and DECTIN-1 (Figure 5D). In sum, our data suggest that the autophagy activation in the force-loaded PDLSCs induces M1 macrophage polarization through the inhibition of the AKT signaling pathway.

## Discussion

Autophagy is an important protective cellular process to maintain cell and tissue homeostasis under mechanical load (Marino et al., 2014). However, it is still unclear that whether and how autophagy influences bone remodeling under mechanical force stimuli. In this study, we showed a novel mechanism that force-induced autophagy in PDLSCs contributed to M1 macrophage polarization, therefore promoting bone remodeling and tooth movement. First, force loading induced autophagy on the compression side of the periodontal tissues during tooth movement, accompanied by the accumulation of M1 macrophages. Blockage of autophagy by 3-MA decreased the tooth movement distance and suppressed M1 macrophage polarization. Second, compressive force *in vitro* stimulated autophagy in PDLSCs, which further increased the expressions of M1 macrophage-related inflammatory elements. These effects could be suppressed or enhanced by the application of autophagy inhibitor 3-MA or autophagy activator rapamycin. Finally, the AKT signaling pathway participated in the regulation of PDLSC autophagy on macrophage polarization under mechanical stimuli.

Autophagy has been demonstrated playing a two-sided role in inflammation and immune responses. On one hand, autophagy in the epithelium decreases the chronic intestinal inflammation (Pott et al., 2018). Depletion of autophagy gene *Atg16l1* in the T cells aggravates the spontaneous intestinal inflammation (Kabat et al., 2016). On the other hand, induction of intestinal autophagy may increase the severity of inflammatory bowel diseases (Zhang et al., 2018). Psychosocial stress may also enhance intestinal autophagy and increase the M1/M2 macrophage ratio in the remaining colon, thus aggravating the severity of inflammatory bowel diseases (Wang et al., 2019). Moreover, higher level of autophagy has been found in the inflammatory periodontal tissues compared with normal periodontal tissues (An et al., 2016). During the force-induced tooth movement process, aseptic inflammatory microenvironment is induced in the periodontal tissues (Garlet et al., 2007). Here, our data showed that autophagy induced by mechanical force participated in the induction of inflammation in the periodontal tissues, which was characterized by the accumulation of M1 macrophages and the elevated expressions of M1 macrophage-related pro-inflammatory cytokines. Furthermore, blockage of autophagy level by 3-MA administration decreased the number of M1 macrophages, bone remodeling and tooth movement process. Consistent with the previous studies, the present study suggests that force-induced autophagy in PDLSCs promotes inflammation in the periodontal tissues by activating M1 phenotypes, thus contributing to bone remodeling and tooth movement.

The role of force-induced autophagy on osteoclast activity is controversial. Previous study has been shown that blockage of autophagy by 3-MA increased the expression of osteoclasts, decreased bone density and promoted tooth movement in the mouse model (Chen et al., 2019). The above findings varied different from our data. The underlying reasons can be interpreted as follows. In the previous study, the trifurcation area was detected for bone density. However, both compression force and strain existed in the trifurcation area, which may influence the assessment of bone density. Moreover, different dosage of 3-MA was used in the present study, which may lead to different outcomes. Nevertheless, further studies on knockout mice should be developed to confirm the regulatory role of autophagy in PDLSCs on macrophage polarization under mechanical force stimuli.

During inflammation and tissue remodeling process, MSCs could interact with immune cells such as macrophages by expressing cytokines such as transforming growth factor-β and secreting elements such as exosomes (Liu et al., 2019; Xu et al., 2019). In the periodontal tissues, PDLSCs are one type of unique MSCs, which can interact with other cells including osteoclasts and immune cells under mechanical stimuli (Zhang et al., 2016; Liu et al., 2017). Previously, we have shown that PDLSCs could secret inflammatory cytokines or gas molecule such as hydrogen sulfide and influence macrophage polarization under mechanical stimuli (He et al., 2015b, 2020). Moreover, autophagy in PDLSCs under compressive force was found to be regulated by lncRNA FER1L4 and involved in tissue remodeling (Huang et al., 2021). In this study, we show a novel mechanism that autophagy in PDLSCs could participate in regulating M1 macrophage polarization under mechanical stimuli, and thus contribute to the bone remodeling process. Given the important roles of MSCs and macrophages during tissue regeneration process, these findings indicate that modulating MSCs autophagy may influence immune cells and therefore contribute to the tissue regeneration process.

We also verify a novel signaling pathway of M1 phenotype activation under mechanical stimuli. Previous studies have shown that several signaling pathways, including β-catenin and STAT-1, are involved in the inflammatory bone remodeling process under mechanical force stimuli (Mao et al., 2018; He et al., 2020). In this study, we find that autophagy in PDLSCs influences M1 macrophage polarization through suppressing the AKT signaling pathway, which is a critical mediator in macrophage polarization (Vergadi et al., 2017). Our data shed light on a novel mechanism of how autophagy regulates macrophage polarization under mechanical force in mechanical stimuli microenvironment.

**FIGURE 6:**
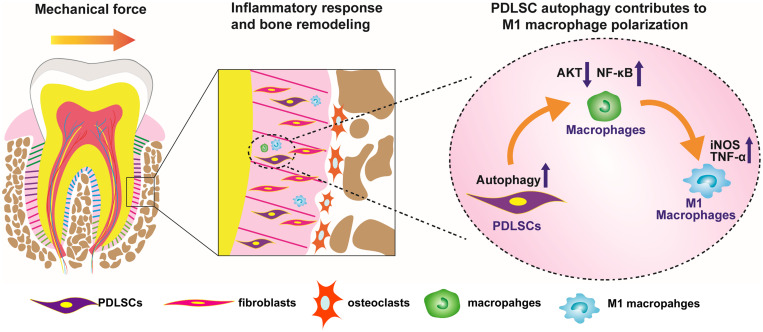
Scheme of the study. Force-stimulated autophagy in PDLSCs steers macrophages into M1 phenotype, which contributes to the bone remodeling and tooth movement process.

In conclusion, force-stimulated autophagy in PDLSCs steers macrophages into the M1 phenotype via the inhibition of the AKT signaling pathway, thereby contributing to bone remodeling and tooth movement (Figure 6). These results lead to a better understanding of how PDLSCs response to mechanical stimuli and interact with macrophage polarization, therefore modulate alveolar bone remodeling. The findings also indicate a possibility that modulating MSC autophagy may regulate inflammatory bone remodeling and regeneration process.

## Data Availability Statement

The original contributions presented in the study are included in the article/Supplementary Material, further inquiries can be directed to the corresponding authors.

## Ethics Statement

The studies involving human participants were reviewed and approved by the Peking University Ethical Committee (PKUSSIRB-201311103). The patients/participants provided their written informed consent to participate in this study. The animal study was reviewed and approved by Institutional Animal Use and Care Committee of the Peking University (LA2013-92).

## Author Contributions

NJ and DH performed the experiments, analyzed the data, and prepared the manuscript. YL and HY designed the experiments, analyzed the data, and prepared and revised the manuscript. YM, JS, XW, SC, ZL, and YZ analyzed the data and revised the manuscript. All authors reviewed the manuscript.

## Conflict of Interest

YM is now employed by the company Beijing Tason Biotech Co. Ltd. The remaining authors declare that the research was conducted in the absence of any commercial or financial relationships that could be construed as a potential conflict of interest.
