# Supplementary material for: Force-Induced Autophagy in Periodontal Ligament Stem Cells Modulates M1 Macrophage Polarization via AKT Signaling
Source: Front Cell Dev Biol. 2021 May 26;9:666631. doi: 10.3389/fcell.2021.666631 (PMC8187804; doi:10.3389/fcell.2021.666631)
Supplement: Supplementary file 1 [file Data_Sheet_1.pdf]

Supplementary Material

1.1 Supplementary Figures

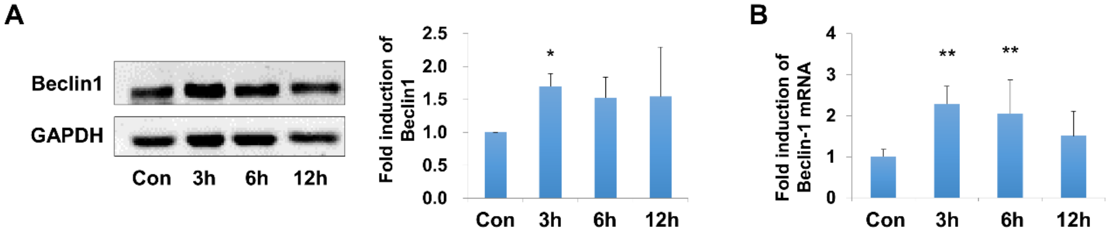

Supplementary Figure 1. (**A**) Western blot of the expression of Beclin1 in PDLSCs under force stimuli (1.5 g/cm^2) at different time points. GAPDH served as an internal control for equal loading. The relative fold change was quantified. (**B**) Relative mRNA expression of autophagy related gene Beclin1 at different time points. Results were presented as mean ± SD. *P < 0.05, **P < 0.01 versus Con.

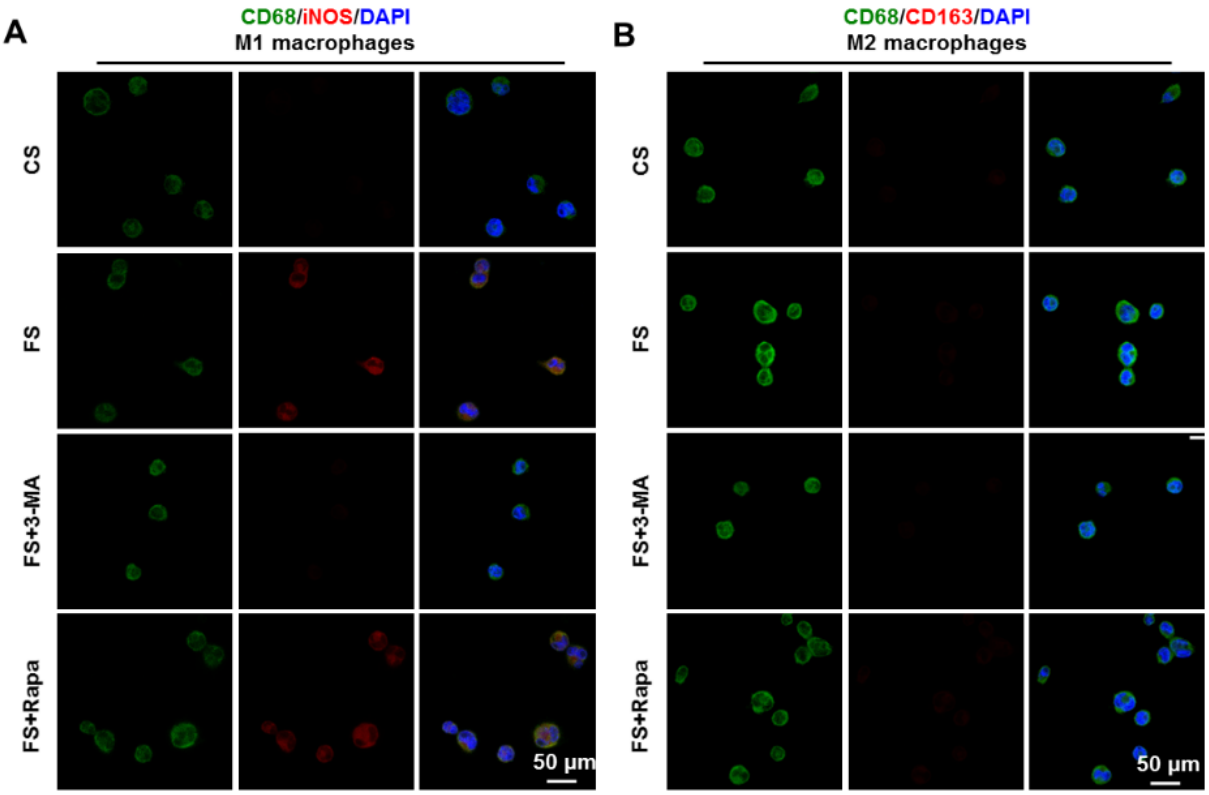

Supplementary Figure 2. Split immunocytochemical images of THP-1-derived macrophages treated with different conditioned medium of PDLSCs (presented in Figure 3C). (A) M1 macrophage polarization was identified by CD68+ (green) and iNOS+ (red). (B) M2 macrophage polarization was identified by CD68+ (green) and CD163+ (red).

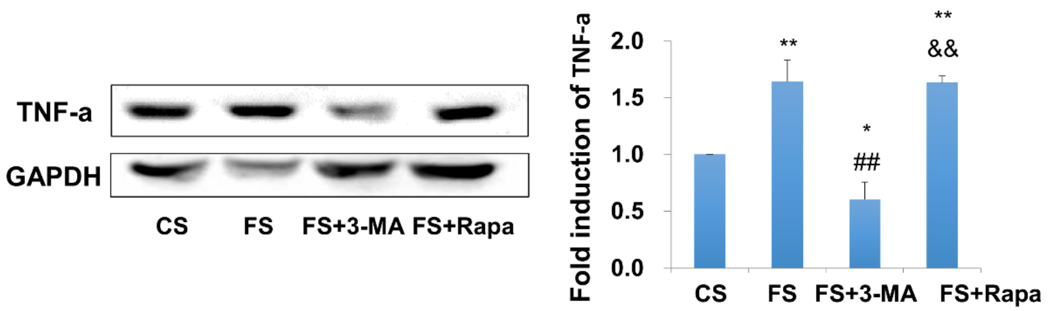

Supplementary Figure 3. Western blot of the expression of TNF-α in THP-1-derived macrophages with treatment of different conditioned medium from PDLSCs. CS: control supernatant; FS: force-loaded supernatant. FS+3-MA: force-loaded supernatant with 3-MA； FS+Rapa: force-loaded supernatant with rapamycin. GAPDH served as an internal control for equal loading. The relative fold change of TNF-α was quantified. Results were presented as mean ± SD. $^{*}P < 0.05$, $^{**}P < 0.01$ *versus* CS; $^{\#\#}P < 0.01$ *versus* FS; $^{\&\&}P < 0.01$ *versus* FS+3-MA.

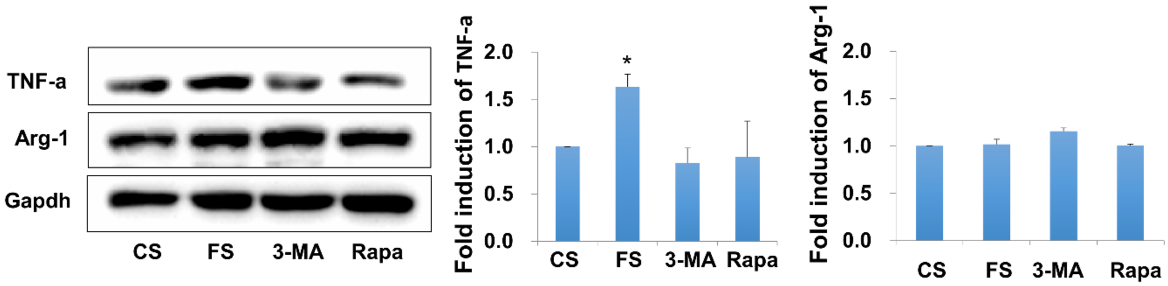

Supplementary Figure 4. Western blot of the expressions of TNF-α and Arg-1 in THP-1-derived macrophages with treatment of different conditioned medium from PDLSCs. CS: control supernatant; FS: force-loaded supernatant. The relative fold changes of TNF-α and Arg-1 were quantified. Results were presented as mean ± SD. **P* < 0.05 *versus* CS.

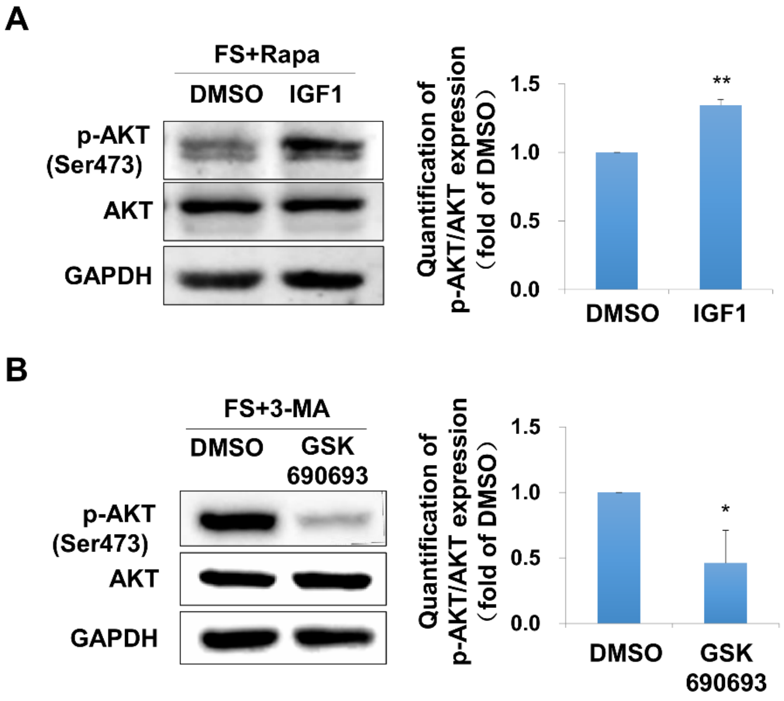

Supplementary Figure 5. Western blot of the expressions of phosphor-AKT (Ser473) and AKT in THP-1-derived macrophages. (**A**) Force-loaded supernatant with rapamycin in the presence of DMSO or AKT signaling activator IGF1 was applied into macrophages. GAPDH served as an internal control for equal loading. The relative fold change of p-AKT/AKT was quantified. (**B**) Force-loaded supernatant with 3-MA in the presence of DMSO or AKT signaling inhibitor GSK690693 was applied into macrophages. GAPDH served as an internal control for equal loading. The relative fold change of p-AKT/AKT was quantified. Results were presented as mean ± SD. *$P < 0.05$, **$P < 0.01$ *versus* DMSO.
